# Characterization of cisplatin-loaded chitosan nanoparticles and rituximab-linked surfaces as target-specific injectable nano-formulations for combating cancer

**DOI:** 10.1038/s41598-021-04427-w

**Published:** 2022-01-10

**Authors:** Muhammad H. Sultan, Sivakumar S. Moni, Osama A. Madkhali, Mohammed Ali Bakkari, Saeed Alshahrani, Saad S. Alqahtani, Nabil A. Alhakamy, Syam Mohan, Mohammed Ghazwani, Haitham A. Bukhary, Yosif Almoshari, Ahmad Salawi, Meshal Alshamrani

**Affiliations:** 1 Department of Pharmaceutics, College of Pharmacy, Jazan University, Jazan, Saudi Arabia; 2 Pharmacology and Toxicology Department, College of Pharmacy, Jazan University, Jazan, Saudi Arabia; 3 Pharmacy Practice Research Unit, Clinical Pharmacy Department, College of Pharmacy, Jazan University, Jazan, Saudi Arabia; 4 Department of Pharmaceutics, Faculty of Pharmacy, King Abdulaziz University, Jeddah, Saudi Arabia; 5 Center of Excellence for Drug Research and Pharmaceutical Industries, King Abudlaziz University, Jeddah, Saudi Arabia; 6 Mohamed Saeed Tamer Chair for Pharmaceutical Industries, King Abudlaziz University, Jeddah, 21589 Saudi Arabia; 7 Substance Abuse and Toxicology Research Centre, Jazan University, Jazan, Saudi Arabia; 8 School of Health Sciences, University of Petroleum and Energy Studies, Dehradun, Uttarakhand India; 9 Department of Pharmaceutics, College of Pharmacy, King Khalid University, Abha, Saudi Arabia; 10 Department of Pharmaceutics, College of Pharmacy, Umm Al-Qura University, Mecca, Saudi Arabia; 11 Department of Pharmaceutics, College of Pharmacy, Jazan University, P.O. Box 114, Jazan, 45142 Kingdom of Saudi Arabia

**Keywords:** Cancer, Nanoscience and technology

## Abstract

The present study was carried out to develop cisplatin-loaded chitosan nanoparticles (CCNP) and cisplatin-loaded chitosan nanoparticle surface linked to rituximab (mAbCCNP) as targeted delivery formulations. The two formulations (CCNP and mAbCCNP) exhibited significant physicochemical properties. The zetapotential (ZP) values of CCNP and mAbCCNP were 30.50 ± 5.64 and 26.90 ± 9.09 mV, respectively; while their particle sizes were 308.10 ± 1.10 and 349.40 ± 3.20 z.d.nm, respectively. The poly dispersity index (PDI) of CCNP was 0.257 ± 0.030 (66.6% PDI), while that of mAbCCNP was 0.444 ± 0.007 (57.60% PDI). Differential scanning calorimetry (DSC) revealed that CCNP had endothermic peaks at temperatures ranging from 135.50 to 157.69 °C. A sharp exothermic peak was observed at 95.79 °C, and an endothermic peak was observed at 166.60 °C. The XRD study on CCNP and mAbCCNP revealed distinct peaks at *2θ*. Four peaks at 35.38°, 37.47°, 49.29°, and 59.94° corresponded to CCNP, while three distinct peaks at 36.6°, 49.12°, and 55.08° corresponded to mAbCCNP. The in vitro release of cisplatin from nanoparticles followed zero order kinetics in both CCNP and mAbCCNP. The profile for CCNP showed 43.80% release of cisplatin in 6 h (R^2^ = 0.9322), indicating linearity of release with minimal deviation. However, the release profile of mAbCCNP showed 22.52% release in 4 h (R^2^ = 0.9416), indicating linearity with sustained release. In vitro cytotoxicity studies on MCF-7 ATCC human breast cancer cell line showed that CCNP exerted good cytotoxicity, with IC_50_ of 4.085 ± 0.065 µg/mL. However, mAbCCNP did not elicit any cytotoxic effect. At a dose of 4.00 µg/mL cisplatin induced early apoptosis and late apoptosis, chromatin condensation, while it produced secondary necrosis at a dose of 8.00 µg/mL. Potential delivery system for cisplatin CCNP and mAbCCNP were successfully formulated. The results indicated that CCNP was a more successful formulation than mAbCCNP due to lack of specificity of rituximab against MCF-7 ATCC human breast cancer cells.

## Introduction

**Figure 1:**
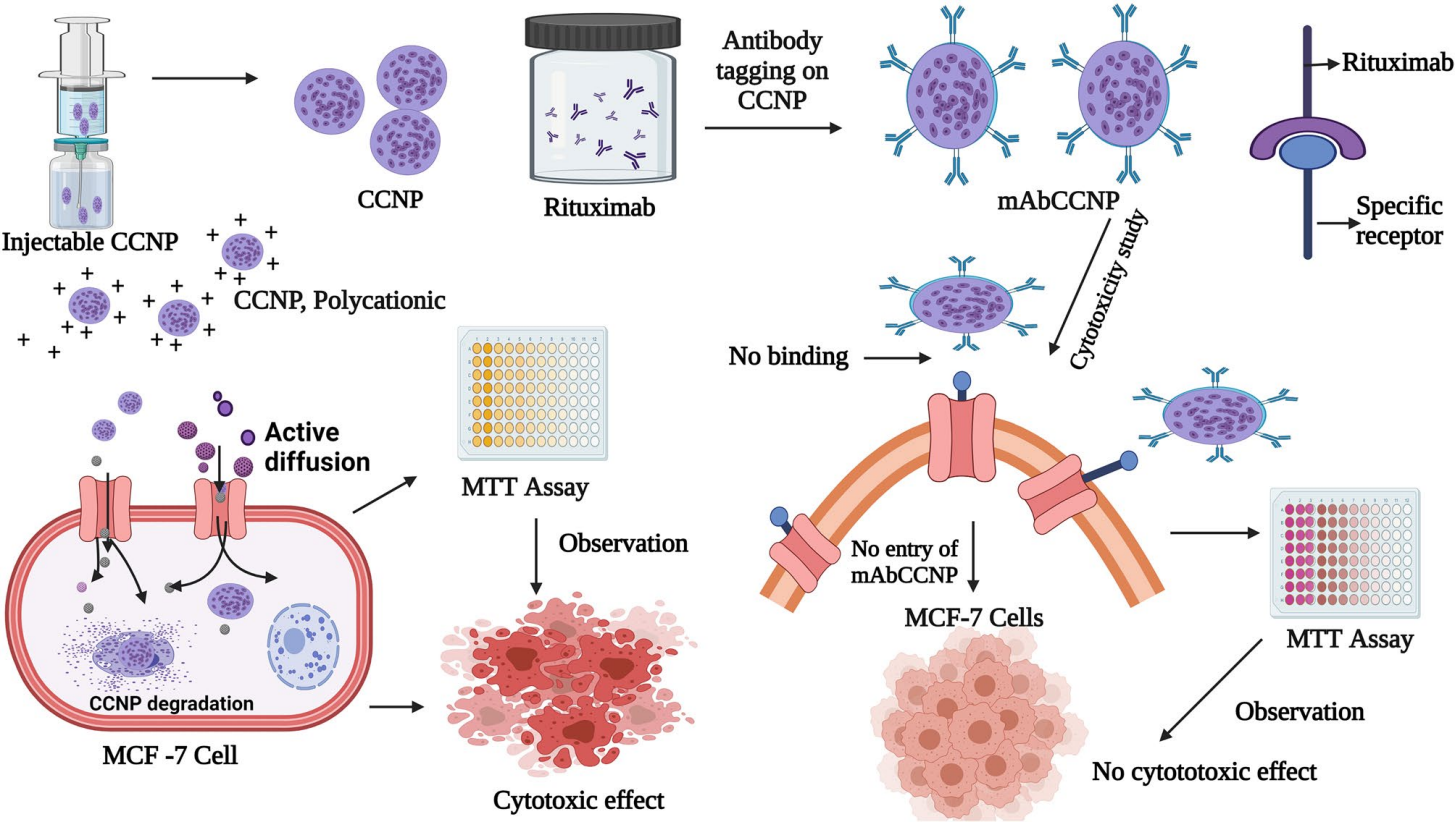
Schematic representation of nanoparticles developed and their mechanistic approach. *CCNP: Cisplatin loaded chitosan nanoparticles, **mAbCCNP: Rituximab surface linked cisplatin loaded chitosan nanoparticles. This figure was Created with BioRender.com, Bio Render, Canada.

Cancer is an invasive disease that leads to high degree of mortality worldwide. The treatment strategies for cancer involve cytotoxic chemotherapeutic agents, radiation therapy and surgery^1^. Drug resistance, which has emerged as the world's biggest health problem, is a major impediment to cancer treatment. Resistance may be caused by inefficient or insufficient delivery of cytotoxic drugs to the cancer cells, resulting in incomplete treatment^2–5^. Cisplatin [cis-diamine platinum (II) dichloride] is widely used as a drug of choice for treating various cancers, and it is the first platinum-based drug approved by US-FDA^6,7^. The mechanism of action of cisplatin involves induction of cytotoxicity in cancer cells and induction of apoptosis. Although cisplatin therapy is successful in various types of cancer, it has been reported that it induces severe nephrotoxicity^8^. Cellular accumulation of cisplatin is directly linked with induction of cytoxicity, intracellular aquation, subcellular distribution, binding to cellular targets, and cellular damage that leads to cell death^9^. Moreover, cisplatin treatment has been associated with therapeutic limitations due to the problem of drug resistance^10^. Drug resistance against cisplatin which leads to treatment failure, is due to multi-drug resistance-associated proteins (MRPs). The development of drug resistance is highly influenced by altered drug transport within cells, changes in glutathione system, induction of apoptotic gene expression, and impaired gene repair mechanism. Targeted nanoparticle drug delivery system has emerged as a promising therapeutic strategy for combating cytotoxic drug-related toxicity, and for overcoming drug resistance^11^. Chitosan polymer has several potential applications in pharmaceutical products since it is non-toxic, and biocompatible and biodegradable; in addition to its high-charge density and muco-adhesion properties^12,13^. Monoclonal antibodies offer the advantages of specificity and good tolerance^14^. Rituximab, a chimeric monoclonal antibody that targets the B-cell marker CD20, has been approved for therapeutic use for humans^15^. In the present study, injectable cisplatin loaded in chitosan nanoparticle surfaces linked to rituximab was formulated and physico-chemically characterized, and its in vitro cytotoxic effect was determined. Since cisplatin is commonly prescribed in the treatment of breast cancer, the in vitro cytotoxicity study was conducted on MCF-7 ATCC human breast cancer cells. The methodologies used in this study and mechanistic approach of CCNP and mAbCCNP are presented as scheme in Fig. 1.

## Materials and methods

### Materials

Cisplatin [cis-diamine platinum (II) dichloride], medium molecular weight chitosan (75% degree of deacetylation, 200–300 cP viscosity grade), tripolyphosphate, monoclonal antibody, RPMI-1640 medium and fetal bovine serum were purchased from Sigma, USA. Chroafil Xtra PVDF syringe filter (0.45-µm) unit purchased from Macherey–Nagel GmbH & Co. KG, Duren, Germany. The solvents used in this study were products of Scharlau, Spain. Ejadah Medical Supplies Est, Riyadh, Saudi Arabia, supplied all items for this study.

### Preparation of cisplatin solution

100 mg of cisplatin was dissolved in 100 mL of Millipore water to make a cisplatin solution. The solution was clarified by heating it for 15 min on a hot plate at 80 °C.

### Preparation of chitosan gel and tripolyphosphate solution

Initially, 1% (w/v) chitosan polymer was prepared in 0.5% (v/v) glacial acetic acid. The chitosan gel (CG) was kept ideally overnight to stabilize. 1% (w/v) tripolyphosphate was prepared in Millipore water and the solution was clarified by heating on a hot plate at 60 °C stirred for 10 min with a magnetic bead.

### Determination of pH and viscosity

The pH was measured using the Oakton pH 700 Benchtop Meter, USA*.* The rheological properties of chitosan gel (CG) were investigated by evaluating the viscosity grade of the CG with a Brookfield viscometer (Model LVDV-E, USA). The samples' viscosity was determined using the Spindle S63. In this process, 50 mL of each solution (CG/CCNP and mAbCCNP) was deposited in a clean and sterile glass beaker. The spindle was dipped into the gel and allowed to settle for 5 min. The viscosity was then determined at room temperature using a 50 rpm rotation speed. The viscosity was measured in centipoise (cP).

### Formulation of cisplatin-loaded chitosan nanoparticles

Cisplatin-loaded chitosan nanoparticles (CCNP) were formulated using an ionic gelation technique. Chitosan polymer is a poly-cationic natural polymer, while tripolyphosphate (TPP) which served as the cross-linker, is anionic in nature. Ionic bonding was achieved via ionic attraction between chitosan and TPP. The CCNP was prepared by stirring 1% (w/v) chitosan gel in a clean and sterile beaker on a hot plate with a magnetic bead at a perpetual speed of 2000 rpm for 10 min. The reaction mixture was stirred at 60 °C for 90 min, resulting in generation of CCNP. During the formulation, cisplatin (0.1%, w/v) and 1% (w/v) TPP were added at pre-determined time intervals. Using a laboratory probe sonicator (CPX ultrasonic processor, Cole Parmer Instruments Co, USA). Sonication was performed at 15 min intervals for 3 min at 100% amplification during the mixing procedure. The product was eluted after 90 min of stirring and kept refrigerated at 2–8 °C for use in subsequent studies.

### Lyophilization process^16^

The CCNP was lyophilized using a Millrock BT85 tabletop freeze dryer, Millrock Technology, USA. Mannitol solution (6% w/v) was combined with the CCNP reaction mixture at a volume ratio of 2:1 in a glass flask. The combination was kept at − 80 °C in a deep freezer for 24 h. Following that, the glass flask was placed in lyophilizing tubes, and the vacuum was induced by opening the knob. The temperature was maintained at − 84 °C, and the vacuum was kept at 3000 Pa (Pascals). After 30 h of lyophilization, the lyophilized nanoparticle powder was eluted from the glass flask, pooled, and kept at + 4.0 °C until used in subsequent study.

### Antibody tagging on cisplatin-loaded chitosan nanoparticles

The lyophilized powder sample of CCNP was subjected to antibody tagging using rituximab. In this technique, 1% (v/v) solution of rituximab was prepared and stirred on a hot plate with a magnetic stirrer. This was designated as reaction mixture (RM). Then, 1% (w/v) CCNP (prepared in Millipore water) was added dropwise to RM at room temperature, with stirring at a constant speed (2000 rpm) for 60 min without heating. The antibody-tagged CCNP (mAbCCNP) was eluted and filtered through 0.45 µm Chroafil Xtra PVDF syringe filter Unit. The mAbCCNP was further lyophilized as described earlier.

### Preparation of samples for analysis

A 1% (w/v) solution of each formulation (CCNP and mAbCCNP) was prepared in Millipore water, and a uniform colloidal solution of formulation was generated by stirring on a hot plate using a magnetic stirrer bead. The injectable colloidal solutions of CCNP and mAbCCNP were filtered using a 0.45-µm Chroafil Xtra PVDF syringe filter Unit. Thereafter, the filtered samples of CCNP and mAbCCNP were subjected to various analysis.

### Physicochemical characterization of nanoparticles

#### Dynamic light scattering (DLS) analysis^16^

The nanoparticles were physically characterized based on zeta potential (ZP) (mV), conductivity (mS/cm), nano size (d.nm and z.d.nm), and polydispersity index (PDI). The dynamic light scattering (DLS) methodology was used to estimate the nanoscale particle size (NS) and polydispersity index (PDI) of each injectable colloidal system. In this study, ZP, NS, and PDI were determined using a Nano-ZS Zetasizer (Malvern Instruments, UK). Each liquid filtrate was placed in a folded capillary cell with no air bubbles in the instrument holder. The colloidal liquid injectable formulations of CCNP and mAbCCNP were characterized using standard procedures in accordance with Malvern Instruments' manual guidelines.

#### Transmission electron microscopy study

The morphological features of CCNP and mAbCCNP were determined using Transmission Electron Microscopy (TEM). This is a technique that produces images of nanoparticles at extremely high resolution. A JEOL JEM-1011 (JEOL USA, Inc, Japan) transmission electron microscope was used to image the CCNP and mAbCCNP samples. Each sample was placed on a carbon-coated grid for TEM, and the instrument was operated at 200 kV. The TEM procedure was carried out according to the method mentioned previously^16^.

#### Differential scanning calorimetry (DSC) analysis^16^

The DSC technique was used to determine the enthalpy changes that occurred as a result of changes in the physical and chemical properties of powdered samples of CCNP and mAbCCNP. The DSC analysis of the CCNP and mAbCCNP was performed using DSC 60 (Shimadzu, Japan). The study was performed in accordance with the method described previously^16^. Each nanoparticle powder sample was placed in an aluminum pan that was not hermetically sealed, and temperature was increased from 30 to 350 °C at the rate of 10 °C per minute, while the atmospheric airflow was maintained at 10 mL min^−1^.

#### X-ray diffraction (XRD) analysis^17^

The crystalline structures of CCNP and mAbCCNP lyophilized powder samples were evaluated using X-ray diffraction (XRD). An XRD analysis of the powdered material was performed using a Unisantis XMD 300 X-ray powder diffractometer (Unisantis Europe GmbH, Germany). The XRD diffractograms were obtained at *2θ* in the range of 2–50° using Cu K α radiation of incident beam (λ = 1.5418 Å) at a voltage of 45 kV and a current of 0.8 mA. A scanning range of *2θ/θ* was selected and scanning speed of 10 min^−1^ was employed.

### Preparation and validation of standard cure

A working stock solution (1000 µg/mL) was prepared by dissolving 10 mg of cisplatin powder in 10 mL of Millipore water. The solution was heated at 80 °C for 10 min. Then, working standard solutions of concentrations 500, 250, 125, 62.5, 31.25 µg/mL were prepared in Millipore water via serial dilution of the stock standard solution in Millipore water. The calibration curve was created by measuring the absorbance of the produced standard dilutions at four different wavelengths and comparing them to a transparent blank (265, 290, 310 and 405 nm) in a UV/visible spectrophotometer. The method was validated by determining linearity at specific wavelengths which indicate consistency with Beer-Lambert’s law. The standard curve was prepared by plotting the absorbance values at λ_max_ against cisplatin concentrations.

### Encapsulation efficacy^18^

Entrapped cisplatin was extracted from 5 g of each lyophilized formulation (CCNP and mAbCCNP) by suspending it in 10 mL of 0.1 N HCl solution in a flask on a hotplate with a magnetic bead for 30 min. The reaction mixture was centrifuged at 2000 rpm and the supernatant was kept at 2 °C. The concentration of cisplatin was extrapolated from the cisplatin standard calibration curve. Then, the encapsulation efficiency (EE) and drug loading (DL) were calculated using the following equations:

$$EE\left(\% \right)=\frac{{\left( {Amount\;of\;drug\;incorporated} \right) - \left( {amount\;of\;free\;drug\;after\;extraction} \right)}}{{\left( {Amount\;of\;drug\;incorporated} \right)}} \times 100$$

$$DL\left( \% \right) = \frac{Total\;amount\;of\;drug\;incorporated\;in\;nanoparticles}{{Total\;weight\;of\;nanoparticles}} \times 100$$

### In vitro release study

Release studies on CCNP and mAbCCNP were conducted in triplicate using dialysis bag (DB). In this procedure, 500 mg of each lyophilized formulation of CCNP and mAbCCNP was placed in a DB individually, and the dialysis bag was immersed separately in 50 mL of phosphate buffer saline, pH 7.4 at 37 °C with a magnetic bead stirring at 1000 rpm for 6 h. The first sampling was done at 30 min to assess burst release phase. Subsequently, 3 mL of the medium was withdrawn every 1 h and replaced with an equivalent volume of fresh medium. The samples were analyzed via UV/visible spectroscopy at 265 nm, and the release pattern was determined by plotting a graph OD against cisplatin concentration.

### In vitro cytotoxicity study

The experiment was performed according to the method developed by Sultan et al.^19^. MCF-7 ATCC human breast cancer cells were grown and maintained in RPMI-1640 with a sodium bicarbonate buffer system (2.0 g/L) at a pH of 7.4 in this process. The media was supplemented with 10% fetal bovine serum (FBS), 100 U/mL penicillin, and 100 µg/mL streptomycin, and cells were cultured individually in a CO_2_ incubator (Heraeus, Germany) at 37 °C, 90% humidity, and 5% CO_2_. The cells were separately treated with 100 μL of varying doses of CCNP (highest dose = 100 μg/mL) and mAbCCNP (highest dose = 500 μg/mL) dissolved in DMSO. 96 well microtiter plates were used for the seeding of cells at a density of 1 × 10^6^ cells/mL (treated and control). The test samples were plated in triplicate (n = 3) and incubated for 48 h in a CO_2_ incubator. Following incubation, each well received 20 µL of MTT at a concentration of 5 mg/mL, and the plates were incubated in the dark for 4 h before the media was removed. The formazan crystals generated in each well were then solubilized with 100 µL of DMSO, and the absorbance of each well was measured at 490 nm in a microtiter plate reader (Biotek ELISA reader, ELX 800, USA). The % cellular viability was estimated once the proper controls were considered. The experiment was performed in triplicate, and the % inhibition of cell propagation was calculated using the formula:

$$Growth\;inhibition\;\left( \% \right) = \frac{{\left( {OD\;control - \;OD\;treated} \right)}}{OD\;control} \times 100$$

### Morphological assessment of apoptosis using double staining method

The effects of cisplatin and CCNP on MCF-7 ATCC cells were evaluated using a double staining technique with acridine orange (AO) and propidium iodide (PI). Following staining, the cells were examined using an inverted fluorescence phase contrast microscope (Nikon Eclipse TE 300, Japan) in accordance with standard procedures^20^. The cells were plated at a density of 1 × 10^6^ cells/mL in a 75 mL culture flask and treated for 72 h with CIS (4.00 and 8.00 µg/mL) and NP (3.50 and 7.00 µg/mL). Following that, the cells were centrifuged for 10 min at 300×*g*. After discarding the supernatant, the cells were washed twice with PBS.

The cell pellets are then treated with 1 µL of AO (10 mg/mL) and 1 µL of PI (10 mg/mL) in equal proportions. The freshly stained cell suspension was put on a glass slide covered with a cover slip and inspected for 30 min after the fluorescence faded using a inverted fluorescence phase contrast microscope (Nikon Eclipse TE 300, Japan). The identification criteria were as follows: (I) viable cells had green nuclei with intact structures; (ii) early apoptotic cells had bright-green nuclei with chromatin condensation; (iii) cells in late apoptosis had thick orange areas of chromatin condensation, while (iv) secondary necrotic cells had orange intact nuclei.

### Statistical analysis

The statistical analysis were done using the Prism 9, Graph Pad Instat software system, USA. Statistical analyses were performed using one-way ANOVA, followed by Tukey’s test (post hoc test). Values of *p* > 0.05, *p* < 0.05,* p* < 0.01 and* p* < 0.001 were considered statistically significant for all analyses.

## Results and discussion

The utilization of chemotherapy with cytotoxic drugs to cure cancer that destroys cancer cells, and noncancerous cells which are present around cancerous cells. Resistance might be due to inefficiently/insufficiently delivering the cytotoxic drugs at the cancer cell site. Thus, the cancer cell develops resistance through various cellular and molecular mechanisms that lead to incomplete treatment. The target-specific drug delivery system can be achieved by using biodegradable nanoparticles surface linked with a monoclonal antibody, a therapeutically more important advancement to treat cancers.

### Physicochemical characterization of nanoparticles

#### Dynamic light scattering analysis

**Figure 2:**
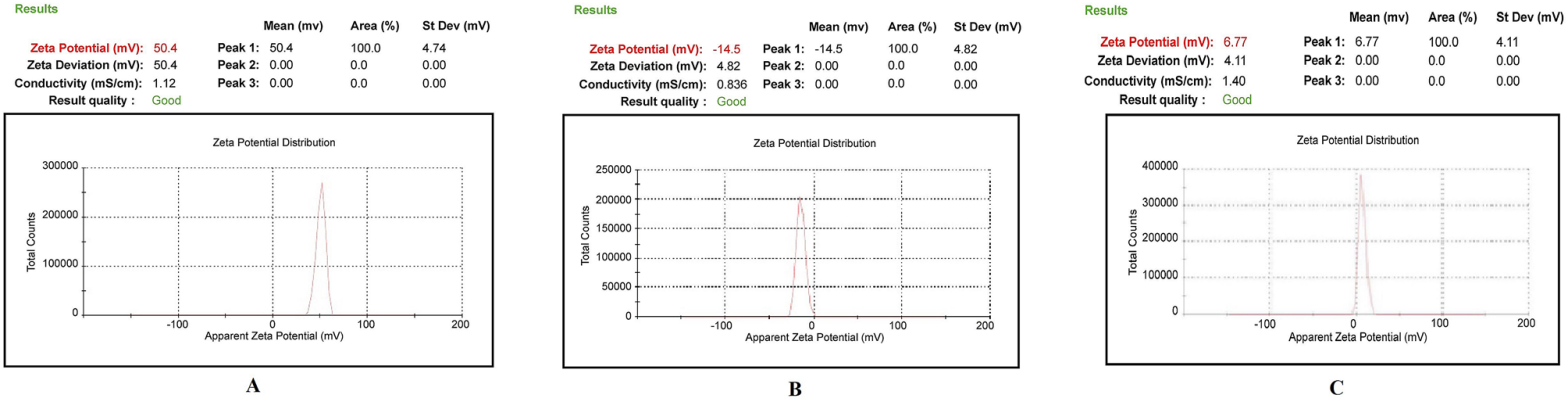
Physical characterization of polymer, cross linker, and monoclonal antibody. (A) Zetapotential analysis of chitosan gel (1.0% w/v). (**B**) Zetapotential analysis of tripolyphosphate (0.5% w/v). (**C**) Zetapotential analysis of rituximab (1.0% v/v).

**Figure 3:**
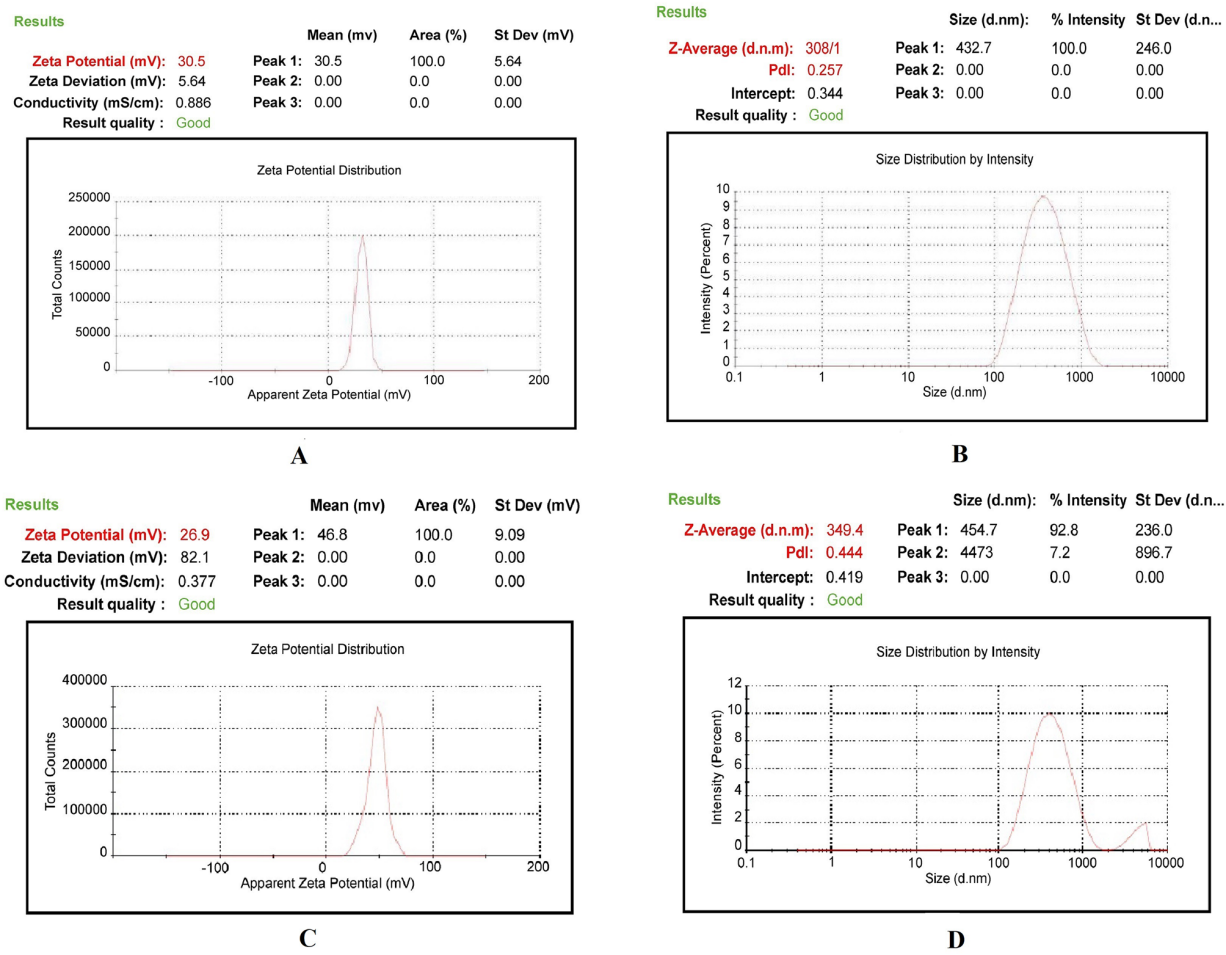
Physical characterization of nanoparticles. (**A**) Zetapotential analysis of injectable CCNP colloidal solution. (**B**) Size distribution analysis of injectable CCNP colloidal solution. (**C**) Zetapotential analysis of injectable mAbCCNP colloidal solution. (**D)** Size distribution analysis of injectable mAbCCNP colloidal solution.

The physical characterization of CG (1% w/v), TPP (0.5% w/v), and rituximab (1% v/v) are shown in Table 1. The results of physical characterization of CG, TPP, rituximab, CCNP, and mAbCCNP are shown in Figs. 2 and 3. The findings are very clear, with unique characteristic peaks which confirmed the unique formulations. The ZP of the CG was + 50.4 mV, indicating the highly cationic and stable nature of the gel (Fig. 2A). An earlier study reported that the ZP of chitosan solution ranged from + 1.65 to + 42.8 mV ^21^. The CG's viscosity grade was estimated to be 131 cP. The ZP value of TPP (0.5% w/v) was − 14 mV (Fig. 2B), and the ZP of rituximab (1% (v/v) was + 6.77 mV (Fig. 2C), which indicates low stability, and their physical properties are summarized in Table 1. The CCNP and mAbCCNP were successfully formulated and their physical characterization are represented in Table 2.

**Table 1:** Physical characterization of polymer, cross linker and monoclonal antibody.

Characteristics	Concentration % w/v	Viscosity grade (cP)	Zeta (mV)	Conductivity mS/cm
Chitosan	1.0	131	+ 50.4	1.12
TPP	0.5	–	− 14.5	0.886
Rituximab	1.0	–	6.77	1.40

*TPP* tripolyphosphate, *cP* centipoise, *mV* milli voltage, mS/cm milli Siemens per centimetre.

**Table 2:** Physical characterization of nanoparticles.

Characteristics	Concentration % w/v	pH	Viscosity grade (cP)	Zeta (mV)	Particle size (zd.nm)	PDI	% Poly dispersity	Conductivity mS/cm
CCNP*	1	5.4	156	30.50 ± 5.64	308.1 ± 1.1	0.257 ± 0.03	66.6	0.686
mAbCCNP**	1	6.2	170	26.9 ± 9.09^#^	349.4 ± 3.2***	0.444 ± 0.007^#^	57.6	0.337

*CCNP: Cisplatin loaded chitosan nanoparticles; **mAbCCNP: Rituximab surface linked cisplatin loaded chitosan nanoparticles; PDI: Poly dispersity index; cP: Centipoise; z.d.nm: Zeta average particle size in nano meter; mV: Milli volt; mS/cm milli Siemens per centimetre. ***Extremely significant at *p* < 0.001when compared to CCNP (mean of n = 3 with standard deviation); ^#^Non significant at *p* > 0.05 when compared to CCNP (mean of n = 3 with standard deviation).

CCNP had a ZP of 30.50 ± 5.64 mV (Fig. 3A), indicating that the nanoparticles were stable. Previous research has revealed that nano formulations with ZPs greater than + 30.00 and − 30.00 mV are more stable^22,23^. The zeta average particle size of CCNP and particle distribution in a colloidal injectable formulation were consistent with the unique parabola depicted in Fig. 3B. The particle size of CCNP was 308.1 z.d.nm, while its PDI was 0.257 ± 0.030. It is worth noting that the parabolic curve and PDI value are indicative of the homogeneity of the CCNP colloidal injectable dosage form. It has been reported that chitosan solid lipid nanoparticles exhibited particle sizes of 110 to 190 nm, as determined through DLS technique. Furthermore, the chitosan solid lipid nanoparticles exhibited ZP of − 26.00 mV, but after coating with chitosan, the ZP value changed to + 22.00 mV^24,25^. The targeting cell is also influenced by nanoparticle size, shape, charge, hydrophobicity, and hydrophilicity. The positively charged nanoparticles would be useful because they will interact favorably with cells with a negative charge that provides more effective transfection actively into the cell^26,27^.

Active targeting can be used in conjunction with passive targeting based on enhanced permeability and retention (EPR) to increase nanomedicine tumor aggregation and retention^28^. Studies suggested that Nanoparticles with sizes ranging from 80 to 300 nm have been found to be beneficial for cancer cell internalization through endocytosis pathways^29–31^. Furthermore, depending on the type of tumor, the pore size of the tumor vessel varies from 200 nm to 1.2 µm^31^. The enhanced permeation and retention (EPR) effect causes the leakiness in tumor vasculatures, which leads to nanoparticle penetration and retention in the tumor bed. The EPR effect is induced by tumor vascular leakiness, allowing nanoparticles to penetrate and remain in the tumor bed. The size of the pores in leaky tumor vasculatures is between 380 and 780 nm. Nanoparticles that are lesser than this threshold can target tumor cells^31,32^. Small particles smaller than 80 nm in size can easily penetrate the tumor by passive diffusion and be pumped back into the bloodstream by the tumor's high interstitial fluid pressure^30,33,34^. The studies also suggested that the size of nanoparticles dictates the pharmacokinetic barriers through which particles bypass and reach target size. According to a previous study, particle sizes ranging from 460 to 2100 nm were associated with increased phagocytic absorption by mouse peritoneal macrophages in vivo^35^.

**Figure 4:**
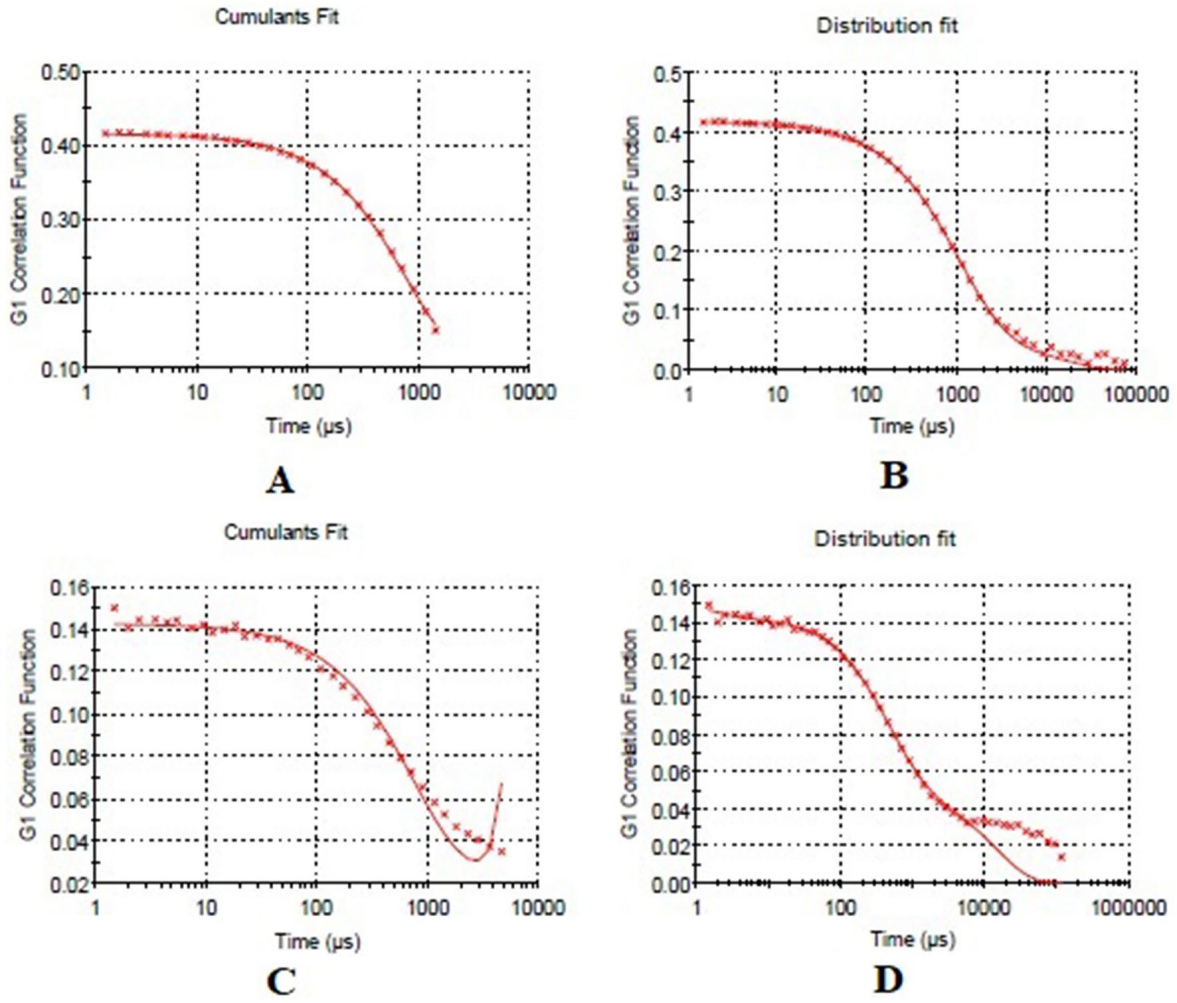
Physical characterization of nanoparticles. (**A**) Cumulative fit analysis of injectable CCNP colloidal solution. (**B**) Size distribution analysis of injectable CCNP colloidal solution. (**C**) Cumulative fit analysis of injectable mAbCCNP colloidal solution. (**D**) Size distribution analysis of injectable mAbCCNP colloidal solution.

Interestingly, when CCNP was coated with rituximab, mAbCCNP exhibited a lower ZP of 26.90 ± 9.09 mV (Fig. 3C) than CCNP, suggesting that the rituximab attachment caused a potential confirmational shift on the surface of CCNP. Furthermore, addition of rituximab to CCNP increased the pH value from 5.4 to 6.2. Interestingly, previous research revealed that increasing the pH of chitosan from 3.0 to 12.0 enhanced its polycationic characteristics^21,36^. Although the ZP of mAbCCNP was lower than that of CCNP, the particles were stable because the ZP was greater than + 25.00 mV. Rong Zhu et al. reported that when α-hederin-entrapped chitosan nanoparticles were surrounded by CD147 monoclonal antibody, the ZP decreased from 20.74 ± 0.75 mV to 10.48 ± 0.79 mV^37^. The study showed that the nanoparticles were round and spherical in shape, with particle sizes ranging from 50 to 300 nm, and PDI of 0.109 ± 0.010. Similarly, in the present study, ZP was decreased from 30.50 ± 5.64 to + 26.90 ± 9.09 mV after coating the surface of CCNP with rituximab (mAbCCNP). The shift of ZP in mAbCCNP was non-significant at *p* > 0.05 when compared to CCNP. The size distribution analysis of mAbCCNP showed that the particle size was increased significantly at *p* < 0.001 from 308.10 ± 1.10 to 349.40 ± 3.20 z.d.nm (Fig. 3D). Furthermore, the polydispersity index was increased from 0.257 ± 0.030 to 0.444 ± 0.007, which was reflected in the particle distribution analysis. It has been reported the particle size of PLGA-NPs increased from 108.30 ± 5.90 nm to 128.40 ± 3.60 nm after conjugation with cetuximab^38^ The short peak representing the distribution analysis of mAbCCNP revealed that 7.2% of the particles were aggregated in colloidal injectable dosage form even after the filtration through a 0.45 µm membrane syringe filter. However, the formulation passed instrumental quality analysis, indicating that the injectable colloidal nanoparticle system was homogenous. The cumulative fit study of the injectable CCNP colloidal solution is presented in Fig. 4A. The CCNP displayed excellent quality with 100% linearity in a colloidal dispersive system. Figure 4B illustrates the 100% linearity of the size distribution fit for CCNP. Figure 4C demonstrates that the mAbCCNP was of high quality in a colloidal dispersive system, with a linearity of 90% and a size distribution fit of 92.80% (Fig. 4D). In a previous work, we found that the formulation of injectable dextran sulfate sodium nanoparticles exhibited 100% linearity in cumulative fit analysis and 90% linearity in size distribution fit^16^. Interestingly, the conductivity of CG (1% w/v) was 1.12 mS/cm (Table 1), which was lowered to 0.686 mS/cm upon CCNP preparation and to 0.337 mS/cm following rituximab coating (Table 2). This shift was reflected in ZP analysis, which suggested that CCNP had a higher electrical conductivity in colloidal system than mAbCCNP.

#### Transmission electron microscopy (TEM)

**Figure 5:**
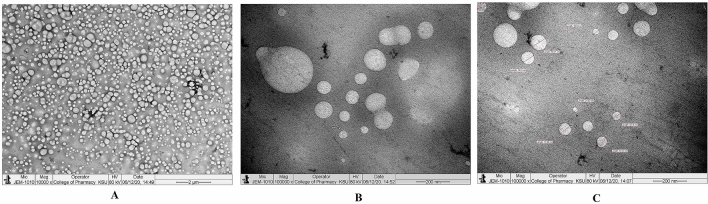
Transmission electron microscope study. (**A**) Morphology of injectable CCNP colloidal solution under ×10,000 magnification. (**B**) Morphology of injectable CCNP colloidal solution under ×100,000 magnification. (**C**) Morphology of injectable CCNP colloidal solution under ×100,000 magnification with size measurement.

**Figure 6:**
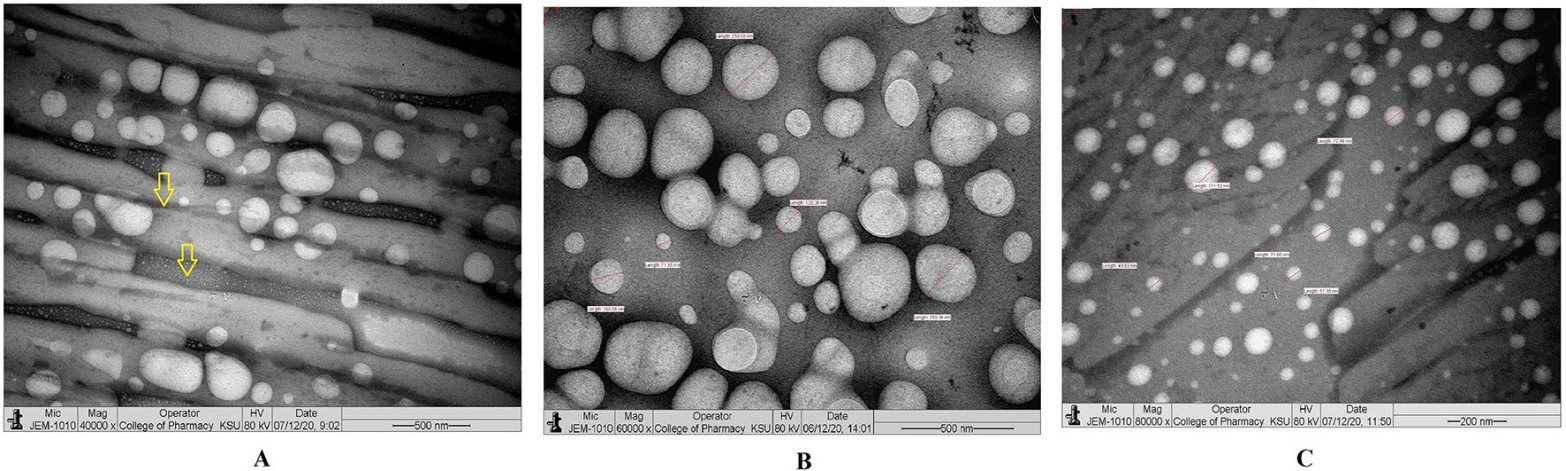
Transmission electron microscope study. (**A**) Morphology of lyophilized CCNP under ×40,000 magnification. The yellow colour aero mark indicating fine lower size nanoparticles embedded in the mannitol powder which is representing the background. (**B**) Morphology of lyophilized CCNP under ×60,000 magnification with size measurement. (**C**) Morphology of lyophilized CCNP under ×80,000 magnification with size measurement.

The TEM images revealed that 1% (w/v) CCNP injectable solution was round and oval-shaped, with smooth surface morphology at various magnifications (Fig. 5A–C). The morphological characteristics of lyophilized CCNP particles are depicted in Fig. 6. The particle sizes were varied and spherical, with smooth morphological features, as shown in Fig. 6A. The lyophilization process caused background clumping of the TEM image. The particles in Fig. 6B,C were at a higher magnification, relative to Fig. 6A. The particles were spherical and smooth, with particle sizes ranging from 50 to 250 nm. An earlier report suggested that TEM analysis of chitosan nanoparticles prepared using TPP as cross linker revealed reduction of mean particle size from 292 to 127 nm. However, another study showed that particle size was highly influenced by degree of deacetylation of chitosan polymer^39^. Interestingly, in an earlier study on chitosan nanoparticles with dextran sulfate, particle sizes of 300 to 1000 nm were obtained, suggesting that the size of chitosan nanoparticles varied with the cross linker used^40^. In that study, TEM analysis showed that the morphology of chitosan nanoparticles cross-linked with dextran sulfate was spherical without drug loading, whereas siRNA-loaded chitosan nanoparticles had rough surfaces and irregular shapes^41^. Figure 6B depicts particles overlapping one another, giving the impression that the particles are surrounded by a zone. In contrast, the particles depicted in Fig. 6C are discrete, with smooth surface characteristics and spherical form. The homogeneous lyophilized CCNP had similar particle morphology and size.

**Figure 7:**
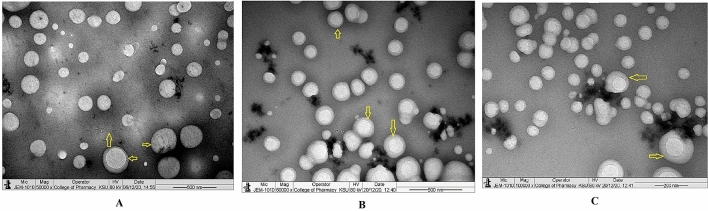
Transmission electron microscope study. (**A**) Morphology of injectable mAbCCNP colloidal solution under ×50,000 magnification. (**B**) Morphology of injectable mAbCCNP colloidal solution under ×60,000 magnification (**C**) Morphology of injectable mAbCCNP colloidal solution under ×100,000 magnification. The yellow colour aero marks of all the three photographs indicating clear zone around the nanoparticle that represents rituximab attachment on CCNP.

**Figure 8:**
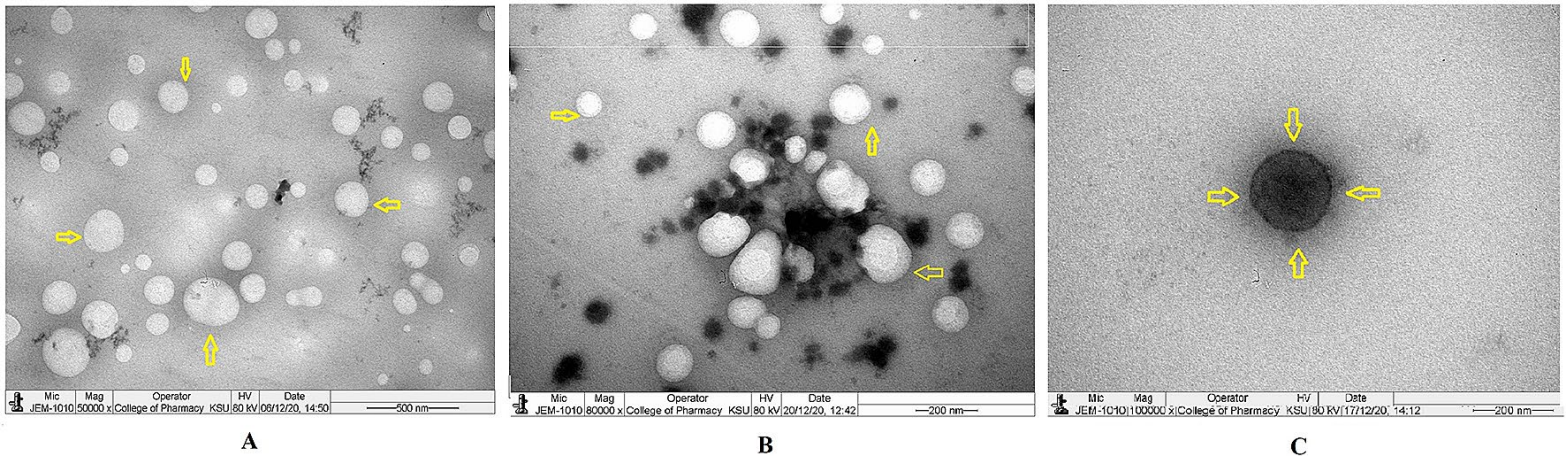
Transmission electron microscope study. (**A**) Morphology of lyophilized mAbCCNP under ×50,000 magnification. (**B**) Morphology of lyophilized mAbCCNP under ×80,000 magnification. (**C**) Morphology of lyophilized mAbCCNP under ×100,000 magnification. The yellow colour aero marks of all the three photographs indicating clear zone around the nanoparticle that represents rituximab attachment on CCNP.

The injectable dosage form of rituximab tagged CCNP (mAbCCNP) is presented in Fig. 7. The mAbCCNP was spherical in shape and homogeneous, with a clear zone around the particles showing mAb surface attachment on CCNP (Fig. 7A). In an earlier report, TEM analysis of α -Hed-CS-CD147-NPs revealed smooth, round, and solid spheres^42^. Recently, a research paper reported that TEM images of unconjugated PLGA-NPs, and PLGA-NPs conjugated with antibodies presented smooth morphology and average particle sizes of 259.28 and 266.40 nm, respectively^38^. Spherical particles are depicted in Fig. 7B, with a surrounding label of antibody. At a higher magnification of 100,000×, the particles showed uniform mAb labeling (Fig. 7C). According to the findings, the homogeneous formulation had uniform size and shape. The lyophilized mAbCCNP (Fig. 8) was discrete in nature, homogeneous, with uniform particle sizes (Fig. 8A,B). The morphological characterization of a single mAbCCNP at a magnification of 100,000× is presented Fig. 8C. The dense layers around the particles indicate the attachment of mAb around each particle, as seen in Fig. 8C. It is clear from the analysis that mAbCCNP was formulated in an injectable dosage form with uniform particles and homogeneous texture.

#### Differential scanning calorimetry (DSC) analysis

**Figure 9:**
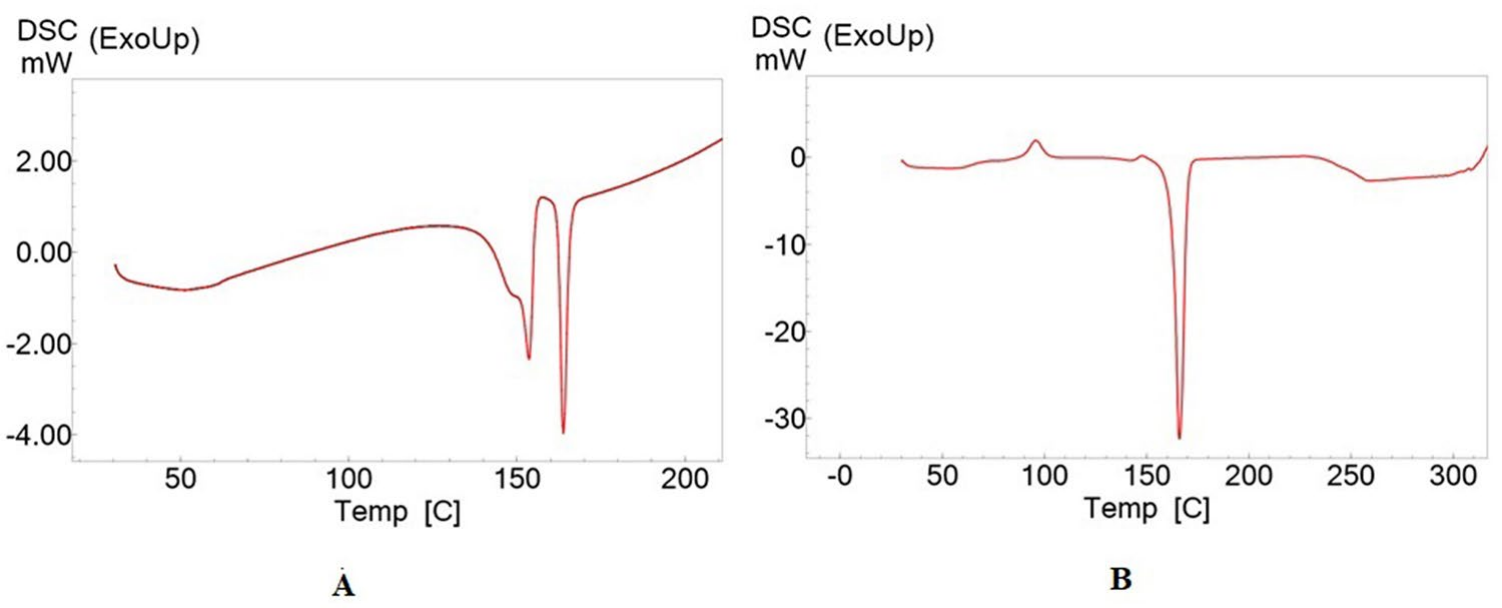
Differential Scanning Calorimetry analysis. (**A**) Thermogram of CCNP. (**B**) Thermogram of mAbCCNP.

The enthalpy changes related to CCNP and mAbCCNP were determined using differential scanning calorimetry, a thermal analytical technique. Figure 9A shows the thermogram depicting the thermal behavior of CCNP. A sharp endothermic peak was observed between 135.50 and 157.69 °C, indicating the thermal degradation property of the nanoparticles. The thermal behavior of mAbCCNP depicted a sharp exothermic peak at 95.79 °C, indicating the protein rituximab attachment on the surface of CCNP, while an endothermic peak observed at 166.60 °C showed the conformational change after rituximab attachment (Fig. 9B). According to a prior study, chitosan has a notable endothermic peak at 152.20 °C and an exothermic peak at 301.10 °C. TPP exhibited an endothermic peak at 139.60 °C in a prior work, while nanoparticles containing ciprofloxacin exhibited two endothermic peaks at 160.40 °C^41^. The differential scanning calorimetry thermogram of chitosan revealed an endothermic peak at 180 °C and an exothermic peak at 370 °C, according to Parisa Yousefpour et al.^42^. An earlier study reported that chitosan nanoparticles prepared using TPP as a cross-linker was stable up to 80 °C^18^. In contrast, the present study demonstrated that CCNP and mAbCCNP were thermostable since their endothermic peaks were observed at 157.69 and 166.60 °C, respectively.

#### X-ray diffraction (XRD) analysis

**Figure 10:**
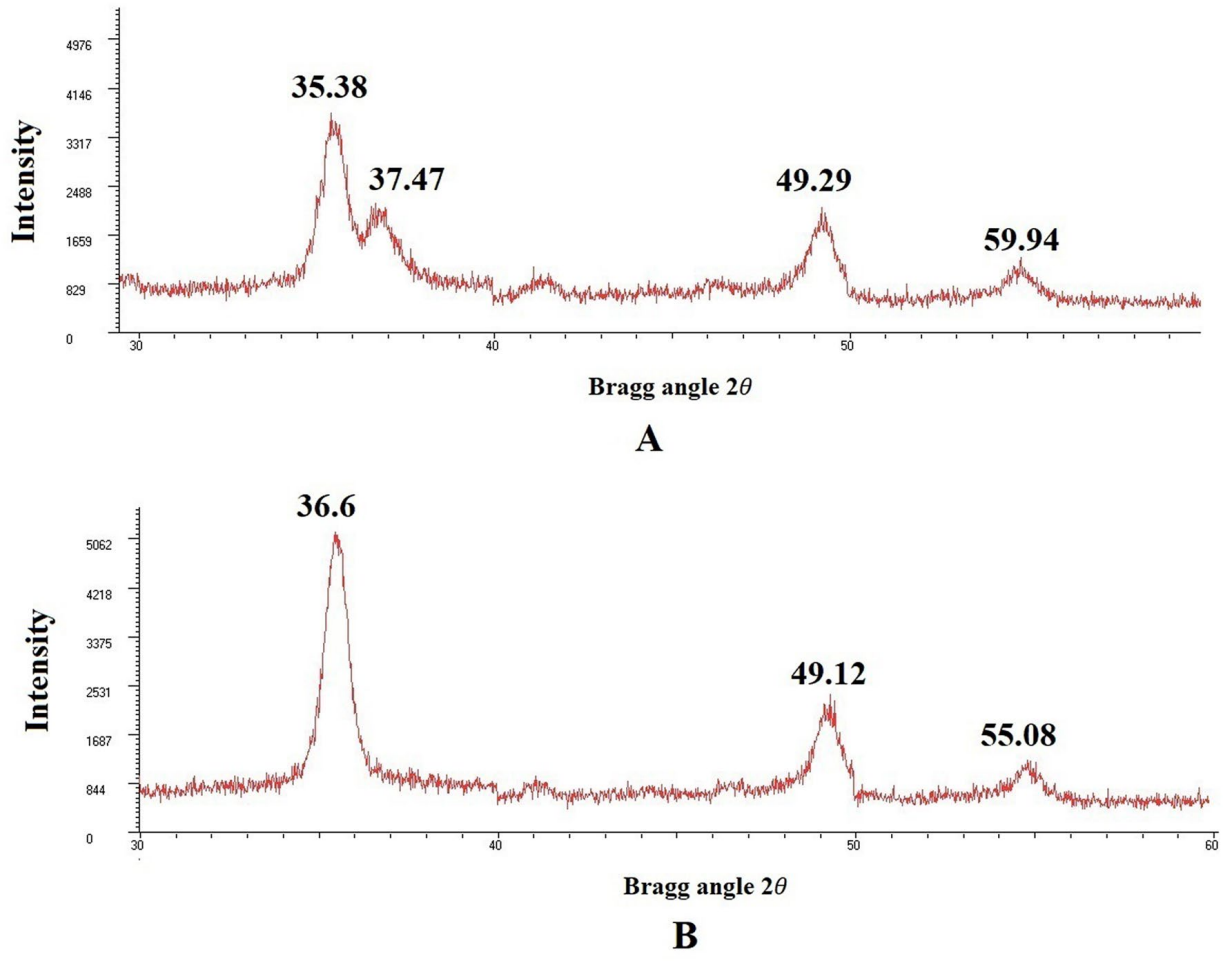
X-ray diffractometer analysis. (**A**) Diffraction pattern of CCNP at *2θ*. (**B**) Diffraction pattern of mAbCCNP at *2θ*.

XRD measurements are used to characterize nanoparticles' distinct crystalline structures. In the current study, XRD analysis at *2θ* revealed distinct nanoparticles based on specific diffraction peaks. The peaks at 35.38°, 37.47°, 49.29°, and 59.94° (Fig. 10A) represent the *2θ* values for CCNP, while the unique peaks at 36.60°, 49.12°, and 55.08° (Fig. 10B) represent *2θ* values for mAbCCNP. In the study by Maria Lazaridou et al., the *2θ* values of CS/TPP/DFO nanoparticles exhibited peaks at 19.32°, 21.03°, 22.56°, 23.98° and 28.40°^18^. Interestingly, in the present study, a broad peak was observed at *2θ* = 35.38° for CCNP, while a broad peak was seen at *2θ* = 36.60° for mAbCCNP. It has been reported the XRD structure of CS–Ag nanocomposite showed unique peak patterns at *2θ* values of 11.70°, 19.80°, 37.90°, 44.00° and 63.90°, indicating the presence of chitosan and silver. In the present study, *2θ* value shifted from 35.38º to 36.60°, 49.29° to 49.12°, and 59.94° to 55.08°, indicating conformational changes in CCNP due to rituximab attachment to its surface.

#### Loading studies and in vitro release profile

**Figure 11:**
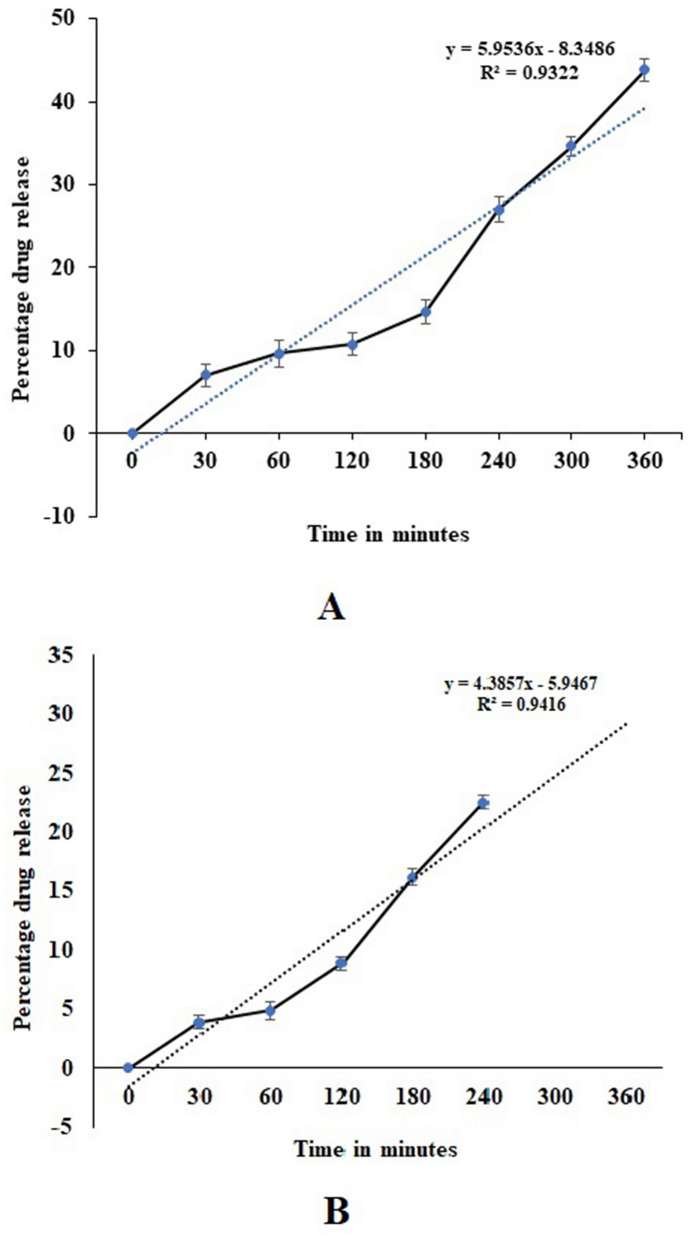
In vitro release profile study. (**A**) Release pattern of cisplatin from CCNP (**B**) Release pattern of cisplatin from mAbCCNP.

Cisplatin was loaded successfully with an entrapment efficiency of 83.3 ± 1.5%, and 92.0 ± 2.0% loading was achieved. The in vitro release profiles of CCNP and mAbCCNP are presented in Fig. 11. It is obvious that the cumulative percentage release of cisplatin from CCNP into the medium was much sustained, with an initial burst release of 7.0% in 30 min (Fig. 11A). The release of cisplatin from CCNP occurred in an irregular manner. The percentage release of cisplatin from CCNP between 60 and 120 min was 1.1%. However, the release between 180 and 240 min was 12.40%, while the release between 300 and 360 was 9.20%. A study of the release profile showed that it followed linearity since the R^2^ value was 0.9322. An earlier report showed 75% release of deferoxamine mesylate from chitosan-TPP nanoparticles in 3 h^18^. In contrast, in this study, there was 43.8% cisplatin released from CCNP in 6 h. Seda and Mehlika reported the release pattern of 5-fluorouracil from chitosan-TPP nanoparticles^43^. Interestingly, the release pattern of 5-fluorouracil demonstrated an initial burst phase range of 12.70–21.20%, followed by total release after 6 h.

The percentage release pattern of cisplatin from mAbCCNP in the release medium is shown in Fig. 11B. Interestingly, the release pattern followed that of CCNP, although the percentage release was lower. On comparing with CCNP, the release of cisplatin from mAbCCNP was 3.90% in 30 min. Similar to CCNP, there was 1% release of cisplatin from mAbCCNP between 60 and 120 min. The release pattern of cisplatin was more or less uniform between 120 and 180 min, and between 180 and 240 min (7.30 and 6.32%, respectively). The release pattern of cisplatin from mAbCCNP was more sustained than the release from CCNP. After 240 min, the release of cisplatin was not significant up to 360 min. However, the release followed linearity (R^2^ value was 0.9416), which was much better than that of CCNP. An earlier study reported that cetuximab-coated thermo-sensitive liposome-loaded doxorubicin showed 70% in vitro release of doxorubicin in 24 h^44^. However, in the present study, cisplatin release was 22.52% in 4 h. Furthermore, the release of cisplatin could not be significantly achieved. This might be due to the blockage of cisplatin from mAbCCNP due to the surface attachment of rituximab. However, there are no existing reports on a similar kind of model.

### In vitro cytotoxicity study

The results of in vitro cytotoxicity study showed that CCNP was able to inhibit the proliferation of MCF-7 ATCC human breast cancer cells. The IC50 value of cisplatin was 2.63 ± 0.95 µg/mL, and CCNP exhibited good cytotoxicity, with IC50 value of 4.085 ± 0.065 µg/mL. The study demonstrated that the minimum amount of cisplatin in sustained release kinetics induced better toxicity than the plain drug molecule. An earlier study reported that cisplatin-entrapped chitosan solid lipid nanoparticle exhibited IC50 value of 1.602 μg/mL against HeLa cells^24^. This might be due to easy passive penetration through the cell membrane pores and slow degradation inside the cells, thereby eliciting sustained action with minimum drug concentration. However, the cytotoxicity of drug also varies with type of cell line. In the present study, CCNP was actively transported inside the cells because chitosan and CCNP are highly polycationic, as was reflected in ZP studies. Oridonin-entrapped galactosylated chitosan nanoparticles (ORI-GC-NP) exhibited IC50 values of 31.07 and 26.59 µM against MCF-7 cells and HepG2.cells, respectively^45^. Furthermore, a study reported that IC50 values of polyethylene glycol-modified titanium dioxide nanoparticles (TiO2 PEG NPs) against HepG2 and A431 cells were 12.00 and 6.00 µg/mL, respectively^46^.

An earlier study indicated that cisplatin induced cytotoxicity in hepatoma cell lines at IC50 and IC90 concentrations^47^. After 48 h of incubation, HepG2 cells were more susceptible to cisplatin than Hep3B cells. According to a report, cisplatin at a concentration of 2.00 µg/mL inhibited cell growth by 37% in HepG2 cells, and 25% in Hep3B cells. The findings indicated that there was no statistically significant difference in IC50 and IC90 values between HepG2 and Hep3B cells. When compared with these results, the formulated CCNP demonstrated a higher level of cytotoxicity. On other hand, mAbCCNP did not exhibit any cytotoxic effect even when the concentration was increased up to 500 μg/mL. Remarkably, in the in vitro release study, cisplatin release from mAbCCNP was 22.52% (8.69 ± 0.23 μg/mL). Surprisingly, mAbCCNP which was rituximab surface-linked CCNP did not elicit cytotoxicity even when the concentration was increased up to 500 μg/mL. This might be due to the specificity of rituximab. Generally, rituximab is a target-specific monoclonal antibody against CD20+ (B cells). The purpose of the research was to develop anticancer injectable dosage formulation against B cell lymphoma. However, as a preliminary step, the study focused on determination of the cytotoxicity against MCF-7 ATCC human breast cancer cells since cisplatin is often prescribed in breast cancer treatment. In contrast, a recent research work^48^ demonstrated that affibody mimics antibody in its ability to specifically target the HER2 receptor. As a result, a HER2 overexpressing cell line, BT474, and a HER2 under-expressing cell line, MCF-7 ATCC human breast cancer cells, were used to assess and compare the targeting of the cisplatin–DNA tetrahedron–affibody to the HER2 receptor. The research demonstrated that a novel cisplatin–DNA tetrahedron–affibody expressed nanodrug with high selectivity targeted HER2-overexpressing breast cancer cells and induced better cytotoxicity when compared to cisplatin. In this study, mAbCCNP was unable to target the MCF-7 ATCC human breast cancer cells, and it failed to induce cytotoxicity.

### Morphological assessment of apoptosis

**Figure 12:**
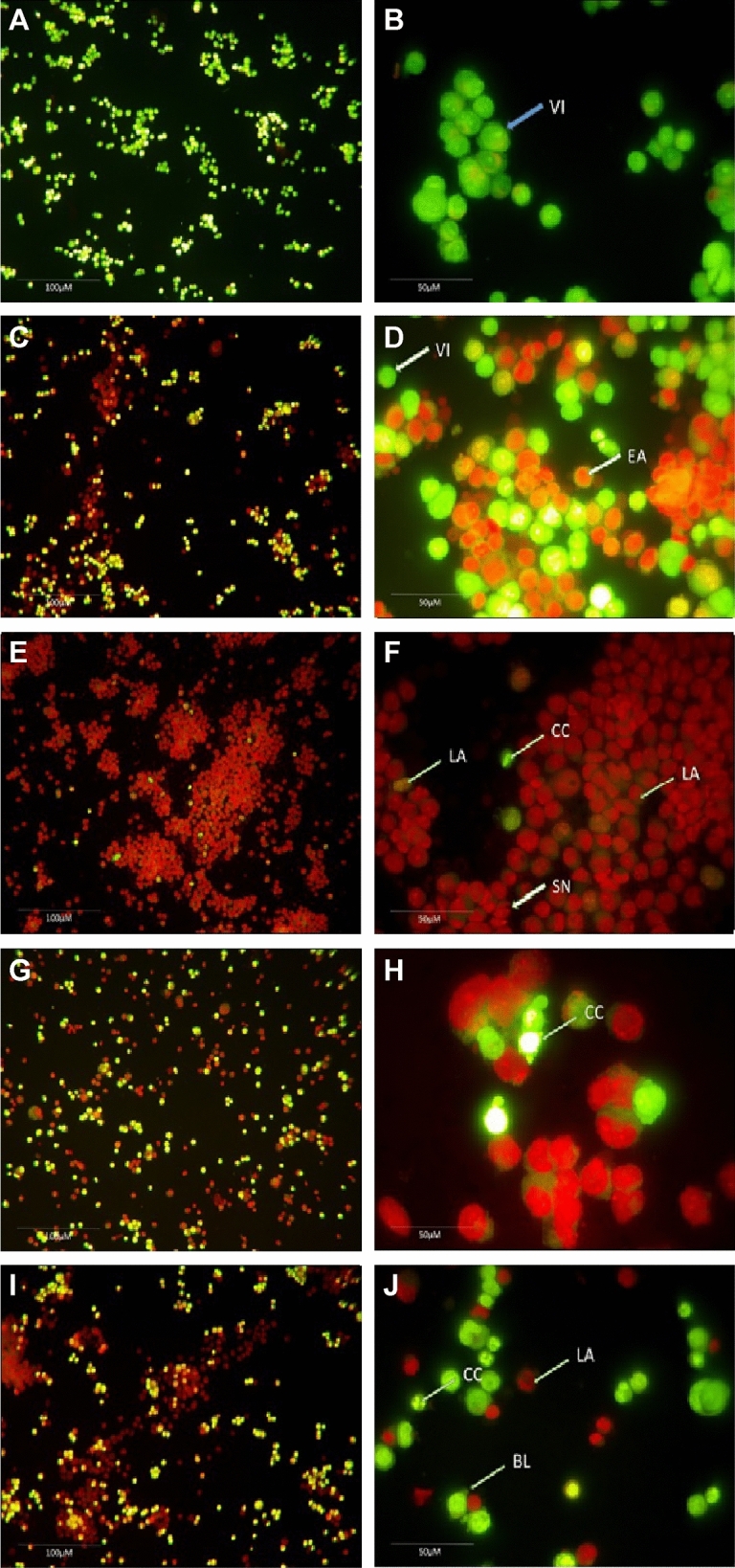
Morphological assessment of apoptotic development of MCF 7 cells by double staining technique using inverted phase contrast microscope. (**A**) Normal viable cells under ×20 magnification. (**B**) Normal viable cells under ×40 magnification. (**C**) Cisplatin treated at the concentration of 4 µg/mL under ×20 magnification. (**D**) Cisplatin treated at the concentration of 4 µg/mL under ×40 magnification, the arrow mark showing both viable cells and early development of apoptosis. (**E**) Cisplatin treated at the concentration of 8 µg/mL under ×20 magnification. (**F**) Cisplatin treated at the concentration of 8 µg/mL under ×40 magnification, the arrow mark showing both viable cells and late development of apoptosis, chromatin condensation and secondary necrosis development. (**G**) CCNP treated at the concentration of 3.5 µg/mL under 20 × magnification. (**H**) CCNP treated at the concentration of 3.5 µg/mL under 40 × magnification, the arrow mark showing the chromatin condensation. (**I**) CCNP treated at the concentration of 7 µg/mL under ×40 magnification. (**J**) CCNP treated at the concentration of 7 µg/mL under ×40 magnification, the arrow mark showing the late apoptosis, chromatin condensation and blebbing of the cell membrane. VI: Viable cells; EA: Early Apoptosis; LA: Late Apoptosis; CC: Chromatin condensation; SN: Secondary necrosis; BL: Blebbing of the cell membrane.

Results of morphological assessment of apoptotic changes in MCF-7 ATCC cells are depicted in Table 3. The results showed that CCNP induced apoptosis either at low dose or high dose. The morphological changes due to apoptosis of MCF-7 ATCC cells are depicted in Fig. 12. The study showed that cisplatin at a dose of 4.00 µg/mL exhibited early apoptosis (Fig. 12D). The study found that cisplatin (4.00 µg/mL) caused early apoptosis. In a previous work, apoptosis was reported in HepG2 cells at a lower dose of cisplatin (4.00 µg/mL) compared to 8.00 µg/mL in Hep3B cells^47^.

**Table 3:** Apoptosis study on nanoparticle treatment.

Group	Concentration (µg/mL)	Viable cells (%)	Early apoptosis (%)	Late apoptosis (%)	Secondary necrosis (%)
Control	–	86.33 ± 4.3	6 ± 2.0	4 ± 2.0	3.66 ± 1.0
Cisplatin	4.0	60.33 ± 4.5	28.66 ± 2.3***	6.66 ± 3.0***	4.33 ± 0.5^ns^
Cisplatin	8.0	20.66 ± 3.5^$$^	26.66 ± 2.8***	39.33 ± 2.0	13.33 ± 3.5
CCNP	3.5	59.33 ± 8.02^ns^	28.33 ± 5.1^ns#^	9 ± 4.5 ns	3.66 ± 2.0^ns^
CCNP	7.0	23 ± 5.0^ns#^	24.33 ± 6.6**	40.66 ± 6.0***	12.33 ± 5.1^ns#^

Each value is the mean of 3 batches (n = 3) with a standard deviation by performing one-way ANOVA followed by Tukey’s test (post hoc test).

^ns^ Non-significant when compared to control sample at *p* > 0.05; ^ns#^ Non-significant when compared to cisplatin concentration of 8.0 µg/mL at *p* > 0.05; ^$$^ Significant when compared to when compared to cisplatin concentration of 4.0 µg/mL and control sample at *p* < 0.001; **Significant when compared to control sample at *p* < 0.01; ***Significant when compared to control sample at *p* < 0.001; CCNP: Cisplatin loaded chitosan nanoparticles.

In the present study, late apoptosis, chromatin condensation, and secondary necrosis were observed when the cells were treated with cisplatin at a dose of 8.00 µg/mL (Fig. 12F). On the other hand, cells treated with CCNP at a dose of 3.50 µg/mL showed chromatin condensation. However, treatment with CCNP at a dose of 7.00 µg/mL resulted in late apoptosis, chromatin condensation, and secondary necrosis (Fig. 12J). Recently, cisplatin and cisplatin-loaded NP were recently evaluated at 10.00 and 30.00 µM concentrations^49^. In their study, the morphology of cells treated with free cisplatin differed significantly from that of cells treated with cisplatin-loaded nanoparticles, with irregular forms, shrinkage, rounding, and detachment in the cisplatin-treated samples. A comparison revealed that samples treated with cisplatin-loaded nanoparticles had reduced degree of cell detachment from the flask bottom and a noticeable shift in cell form, with absence of intracellular connections. Overall, the present study demonstrated that CCNP as potent chemotherapeutic agent to treat breast cancer.

## Conclusion

The present study has demonstrated successful design and production of nanoparticle formulations of CCNP and mAbCCNP for targeted delivery to cancer cells. The physicochemical characterization of CCNP and mAbCCNP showed significant properties as injectable dosage forms. The CCNP produced significant cytotoxicity on MCF-7 ATCC human breast cancer cells. However, mAbCCNP did not exert cytotoxicity on MCF-7 ATCC human breast cancer cells due to absence of target-specific receptors.
